# Induction of autophagy in one-cell stage somatic cell nuclear transfer embryos improves preimplantation embryonic development in goat species

**DOI:** 10.1371/journal.pone.0314176

**Published:** 2025-04-28

**Authors:** Nasrin Mahvash, Reza Moradi-Hajidavaloo, Farnoosh Jafarpour, Mehdi Hajian, Mohsen Rahimi, Nafiseh Sanei Ata-abadi, Marjan Sadeghi, Mohammad Hossein Nasr-Esfahani

**Affiliations:** 1 Department of Biology, Faculty of Science and Technology, ACECR Institute of Higher Education (Isfahan), Isfahan, Iran; 2 Department of Animal Biotechnology, Reproductive Biomedicine Research Center, Royan Institute for Biotechnology, ACECR, Isfahan, Iran; 3 Department of Biology, Naghshejahan Higher Education Institute, Isfahan, Iran; AllExcel, Inc., Shelton, CT, USA, UNITED STATES OF AMERICA

## Abstract

Autophagy is a lysosome-mediated catabolic pathway that is dependent on the mammalian target of rapamycin (mTOR). It plays a crucial role in the degradation of aged organelles and macromolecules. Several studies have explored the role of autophagy in embryonic genome activation and its significance during the early preimplantation development of mammals. In our study, we showed that autophagy is inhibited in one-cell stage SCNT embryos when compared to fertilized counterparts in goats. Notably, we found that 6-DMAP, a kinase inhibitor, reduces the phosphorylation of ERK1/2.This reduction correlates with a decrease in autophagy levels, as indicated by the presence of LC3 puncta in 6-DMAP treated embryos. To address the inhibition of autophagy in goat SCNT embryos, we induced autophagy using Rapamycin at concentrations of 10 and 100 nM for 6 hours, immediately following chemical activation. This induction led to a significant improvement in the development of goat SCNT embryos, as evidenced by an increased blastocyst rate compared to the control group. Our findings suggest that the induction of autophagy during early hours of one-cell stage embryos is critical for pre-implantation development in goat SCNT embryos warrant further investigation. This research opens new avenues for understanding the role of autophagy in embryonic development and its applications in reproductive biotechnology.

## Introduction

Early embryonic development is a highly sophisticated process. This complexity arises from the unique dynamics of gene expression transitions and the gene regulatory network [1]. Two vital developmental processes that regulate this period are maternal-to-embryonic transition (MET) and embryonic genome activation (EGA) [2]. EGA occurs in a species-specific manner, initiating at the two-cell stage in mice, the four-to-eight-cell stage in caprine, porcine, and humans, and the eight-to-the-sixteen cell stage in ovine and bovine species [3–8]. The gradual degradation of maternal mRNAs and proteins is a critical prerequisite for the MET period [9–14]. Following this degradation, the embryonic genome becomes active, producing exclusive mRNAs and proteins that take control of the developmental processes [13,15–17]. Various mechanisms mediate the degradation of maternal RNAs during the MET period. These mechanisms include the interaction of RNA-binding proteins with 3´-untranslated regions (UTRs) of target RNAs and the interaction of target genes with microRNAs (miRNAs) [18, 19]. The degradation of maternal proteins involves two main pathways: the ubiquitin-proteasome system (UPS) and macroautophagy (referred to as autophagy in this study) [20–22]. Autophagy, also known as “self-eating”, is a mammalian target of rapamycin (mTOR)-dependent, lysosome-mediated catabolic pathway responsible for the degradation of long-lived organelles and macromolecules [23]. This mechanism is crucial for maintaining cell viability and homeostasis under imbalanced conditions [22]. Autophagy is astepwise process orchestratedby several gene products and compromises initiation, expansion, maturation, and fusionstages [24–27].

Among the autophagy-related (ATG) proteins, microtubule-associated protein 1A/1B-light chain 3 (LC3) is particularly crucial. LC3 exists in two forms: LC3-I, a cytosolic form, and LC3-II, a lipidated form that associates with the autophagosomal membrane. The conversion of LC3-I to LC3-II is widely used as a marker for autophagy induction [28]. During autophagy, LC3-II binds to the autophagosomal membrane, facilitating the elongation and closure of autophagosomes, which are then fused with lysosomes for degradation [29]. The presence of LC3-II indicates autophagic activity and is essential for the maturation of autophagosomes. This conversion process depends on several ATG proteins, such as ATG3, ATG4, ATG5, ATG7, and ATG12, which work together to ensure the proper formation and function of autophagosomes [30–32]. In embryonic development, the role of LC3 and other ATG proteins is critical for maintaining cellular homeostasis, especially during the periods of extensive cellular reorganization and differentiation seen in early stages [33].

Several studies are investigating the essential role of autophagy during MET and EGA events, as well as its regulatory role during the early preimplantation development of mammals [13,33,34]. *Atg5*-knockout mouse embryos revealed developmental arrest between the four-cell and eight-cell stages, indicating the regulatory activity of autophagy during these early stages [13,33]. Interestingly, fertilization of *Atg5*-knockout oocytes with wild-type spermpartially bypassed embryonic arrest [13,33]. Thispartial rescue of embryonic development in these mouse embryoswas likely due to delayed autophagy activation, since EGA (including *Atg5* gene activation) occurs at two-cell stage in mouse embryos [33]. Furthermore, some studies have shown that chemical inhibition of autophagy results in developmental arrest at the four-to-eight cell or morula stage [13,35–38]. Additionally, Song et al. demonstratedthat most ATG genes remained epigenetically active and were stably expressed throughout early embryogenesis in humans [39]. Together, these studies underscorethe importance of the autophagy pathway in early embryo development.

Developmental defects insomatic cell nuclear transfer (SCNT) embryosoften occur during EGAacross various species, including cattle, humans, mice,pigs,and sheep [40–42]. A systematicinvestigation of gene expression at single-cell resolution revealed that a group of 300 genes failed to be properly activated in SCNT embryos as compared to their IVF counterparts in mice. Further analysis indicatedthat these genes areenriched in processes such as ribosome biogenesis, transcription, RNA processing, RNA splicing and mRNA transport [43]. These genes may have regulatory effects on autophagy, potentiallyhamperingEGA in developing SCNT embryos.

Based on evidencesuggesting the importance and regulatory role of autophagy in EGA and early embryogenesis, we designed the current study with two main objectives. First, we aimed todecipher the autophagy status in SCNT goat embryos compared to IVF embryos. Second, based on our initial results,we investigated the effectsof autophagy inductionusing rapamycin on the preimplantation development of goat SCNT embryos.Discovering new mechanisms and integrating them into SCNT procedure to improve its efficiency is of great interest and value.

## Materials and methods

### Media and reagents

All media and reagentswere obtained from Sigma Chemical Co. (St. Louis, M0O) and Gibco (Grand Island, NY, USA), unless otherwise specified.

### Ethics and animals

All methods were performed in accordance with the guidelines and regulations of the Institutional Review Board and the Institutional Ethical Committee of the Royan Institute.

Goat ovaries used in this study were obtained from female goats at alocal slaughterhouse in Khomeini Shahr, Isfahan, with the permission of the slaughterhouse manager and the agreement of the veterinary organization.

### Preparation of Rapamycin solution

Commercially available Rapamycin (37094; Merck) was dissolved in DMSO to create a 10 mM stock solution, which was thenstored at -20 °C. The stock solution was later diluted in synthetic oviduct fluid (SOF) to obtain the desired concentrations of 1 nM, 10 nM and 100 nM.

### In vitro maturation (IVM) of goat oocytes

The ovaries were collected and transported to the laboratory, where cumulus-oocyte complexes (COCs) were released from 2–6 mm follicles and graded. Grade 1 and 2 COCs (thosewith homogenous cytoplasm and a minimum of three layers of compact cumulus cells) were selected forprocessing IVM as previously described [44]. The immature COCs were then cultured for 24 hours in IVM medium containing luteinizing hormone (LH) (10 mg/mL), follicle stimulating hormone (FSH) (10 mg/mL), 17 β-estradiol (E2) (1 mg/mL), cysteamine (0.1 mM), insulin-like growth factor 1 (IGF1) (100 ng/mL) and epidermal growth factor (EGF) (100 ng/mL) [45]. Following this initial culture period, The COCs were placed in groups of 10 within a droplet of IVM medium (50 µl) under mineral oil in a 60-mm polystyrene dish. The cultures were maintained at 38.5 °C in a humidified atmosphere of 5% CO_2_, conditions that are critical for promoting optimal oocyte maturation.

### Invitro fertilization (IVF)

The matured and cumulus-expended COCs were subjected to the IVF procedure as previously described in our previous studies [46]. To isolate motile sperm from non-motile sperm in freeze-thawed samples, the Swim-Down method was employed, which involves layering the sperm over a density gradient and allowing motile sperm to swim down into the medium. Subsequently, 1 x 10^6^/mLmotile sperms were introduced into droplets (50 µl) of fertilization medium containing 10 matured COCs. The co-incubation of matured COCs with motile sperms occurredfor 20 h at 38.5 ºC in a humidified atmosphere with 5% CO_2_, conditions that are critical for optimizing fertilization rates.

### In vitro culture (IVC)

After 18–20 h ofco-incubation of matured COCs with isolated motile sperm, the cumulus cells were removed from presumptive zygotes by vortexing. Subsequently, presumptive zygotes were cultured in groups of 7 in droplets (20 µl) of SOF culture medium supplemented with glucose, charcoal stripped serum, ITS (insulin, transferrin, and selenium) and myo-inositol (modified SOF: mSOF) for 7 days. The mSOF medium provides a balanced nutrient composition that supports early embryonic development. The presumptive zygoteswere cultured under mineral oil in a 60-mm polystyrene dish at 38.5 °C, with an atmosphere of 5% of CO_2_ and 5% of O_2_ in a humidified incubator. On Day 3 and day 7, the cleavage and blastocyst rates were evaluatedto assess embryo developmental competence.

### Somatic cell nuclear transfer (SCNT)

Handmade SCNT technique was employedin this study for the production of goat SCNT embryos. To this end, after removing the cumulus cells from matured COCs using 300 IU/mLhyaluronidase, the zona pellucida (ZP) was also removed by incubating thedenuded matured oocytes in 5 mg/mLpronase solution for 30–45 seconds. Manual enucleation was performed using a fine hand-pulled Pasteur pipette to remove the oocyte nucleus, a critical step in the SCNT process. Toreduce potentialinjury to the oocytes during manual enucleation, zona free oocytes were treated with 4 μg/mLdemecolcine for 20–30 minutes (min). The procedures forrenucleation, electrofusion, and artificial activation of the reconstructed oocytes were conducted as previously described [47]. Following activation, reconstructed oocytes were cultured in droplets of 6-DMAP supplemented with 0, 1, 10 and 100 nM Rapamycin and incubated at 38.5 °Cin a humidified atmosphere with5% CO2for 4 h. Subsequently, the activated oocytes were transferred to mSOF supplemented with the same concentrations of Rapamycin for an additional 2 h.They were then transferred to mSOF and cultured under similar condition to IVF embryos. Finally, on Day 3 and Day 7, the cleavage and blastocyst rates were evaluated to assess embryo developmental competence.

### Immunofluorescence staining for detection of LC3 in pronuclear stage formation

The presumptive zygotes from IVF at 11, 14, and 17hours post insemination (hpi) were selected for immunostaining to detect LC3. Additionally, SCNT pseudo-zygotes at 3, 6, and 9hours post activation (hpa) were selected for LC3detection. While Comizzoli and colleagues demonstrated that the first S-phase occurs approximately 8 hpi, therefore,we chose 11, 14 and 17 hpi in IVF embryos ascounterparts for 3, 6 and 9 hpa in SCNT embryos [48].The effect of rapamycin on autophagy induction was assessed through LC3 immunostaining in SCNT pseudo-zygotes.

**Fig 1:**
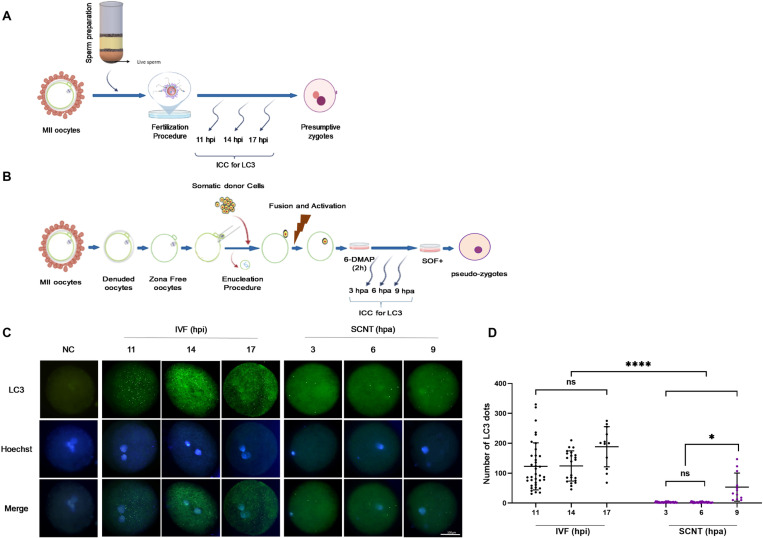
A schematic review of **(A)** IVF and **(B)** SCNT procedures that were carried out in this study for evaluating the expression of LC3 in pronucleus stage. **(C)** Expression of LC3 in the pronucleus stage of IVF (11, 14 and 17 hpi) and SCNT (3, 6 and 9 hpa) goat embryos. Green, LC3 labelled protein (upper row); Blue, chromatin labelled with Hoechst (middle row) and Merge, LC3 (green) and chromatin (blue) merged (lower images) images. Scale bar is the same in all pictures and represents 100 μm. **(D)** Quantification of LC3 in IVF and SCNT goat embryos. *P< 0.05, ****P< 0.0001 and ns, non-significant.

To this end, the zona pellucida was removed from IVF zygotes (the SCNT pseudo-zygotes were already free from the zona due to the handmade SCNT technique).The zygotes and pseudo-zygotes were washed three times in PBS/PVA, fixed in 4% paraformaldehyde for 20 min at room temperature (RT), and washed several times in PBS/PVA. Following fixation, they were incubated in a permeabilization solution (0.5% Triton-X-100) for 30 min at RT, followed by incubation for 1 h in a blocking solution consisting of PBS containing 1% bovine serum albumin (BSA) and 10% goat serum. Next, the zygotes and pseudo-zygotes were incubated overnight at 4°C with LC3 primary antibody (1:200, NOVUSBIO, Nb100–2220) in antibody buffer (PBS/PVA containing 0.1% Triton-X-100 and 1% BSA).After extensive washing, the samples were incubated with goat anti-mouse IgG conjugated with FITC (Millipore; AP124F) for 45 min at 37ºC in the dark. Nuclear labeling was achieved by incubation with 1 μg/ mLHoechst 33342 for 15 min at RT. Finally, the immunostained zygotes and pseudo-zygotes were rinsed in PBS/PVA and placed on a slide containing mounting solution, covered with a coverslip, and observed with a 40x objective using a fluorescence microscope (Olympus BX51, Tokyo, Japan). Appropriate negative controls (NC) for primary and secondary antibodies were included in our immunostaining (Fig 1C, NC).While rapamycin is the only intervention in our study, we could not include a positive control (PC) in the immunofluorescence staining, as treating the embryos with rapamycin serves as the best positive control.The images were captured using a sensitive camera (Olympus DP71) withanalysis performed using LS research software. The number of LC3-positive puncta was quantified by using the count tool in ImageJ (National Institutesof Health, Bethesda, ME, USA, http://imagej.nih.gov/ij/).

### Western blotting

For Western blotting analysis, approximately 200 matured oocytes and 200 pseudo-zygotes were used, both in the presence andabsence of 6-DMAP (2 hpa). The matured oocytes and pseudo-zygotes were lysed using TRIzol™ reagent (Thermo Fisher Scientific, USA) according to the manufacturer’s guidelines. Equal amounts of each protein sample (approximately 30 μg of protein) were separated by SDS-PAGE and transferred to PVDF membranes (Bio-Rad, USA). After blocking the membranes with 10% skim milk (Millipore, USA), theywere incubated with different primary antibodies for 2 h at RT.

The primary antibodies used were rabbit anti-p-ERK antibody (1:50, Cell Signaling, 9101) and anti-β-actin antibody (1:1000, Santa Cruz, sc-47778). Thereafter, the membranes were incubated for 1 h at RT with secondary antibodies, which included horseradish peroxidase (HRP)-conjugated goat anti-mouse IgG (1:50000, Dako, P0447, Denmark) and HRP-conjugated mouse anti-rabbit IgG (1:8000, Santa Cruz, sc2357). The HRP-conjugated IgG bound to each protein band was visualized by Gel Documentation System (UVTEC, Cambridge). The ImageJ software was used to quantify the intensity of each band for evaluation the protein expression.

### Analysis of mRNA expression in blastocysts

The relative expression of developmentally important genes, including caudal type homeobox 2 (*CDX2*), pluripotent POU domain, class 5, transcription factor 1 (*POU5F1*), sex-determining region Y-box 2 (*SOX2*), Nanog Homeobox (*NANOG*), and microtubule-associated proteins 1A/1B light chain 3B (*LC3*) was assessed in blastocysts.

These blastocysts were produced throughSCNT and IVF across three replications. On Day 7,the blastocyst were collected,extensively washed in PBS/PVA and then suspended in groups of 6 in lysis buffer before beingstored at -80ºC.

Total RNA was isolated from expanded blastocysts at Day 7 using the RNeasy Plus Micro Kit (QIAGEN, Germany, 74034) in each replicate. The total RNA was then reverse transcribed using a cDNA Synthesis kit (Biotech rabbit, Germany) according to the manufacturer’s protocol. The quality and integrity of the cDNA were verifiedusing PCR with ahousekeeping primer (*B-ACTIN*) as a reference gene in the RT-PCR analyses. Three technical replicates were performed for each sample, and the mean cycle threshold (CT) was calculated. Relative expression was determinedusing Ct values normalized against *B-ACTIN* and the 2^-ΔΔCT^ equation was employed to quantify changes in gene expression. All the primers were designed using thePrimer 3 program (https://primer3.ut.ee/) and their characteristics are listed in S1 Table.

### Statistical analysis

Continuous variables were analyzed using GLM procedure after testing for normality (Shapiro-Wilk) using UNIVARIATE procedure. Tukey Studentized Range (HSD) test was used for pair-wise comparisons. If the assumptions of parametric tests were violated, data were analyzed using Kruskal-Wallis test. Data with discrete nature were analyzed using GENMOD procedure including logistic regression (log) as Link Function and Binomial as type of distribution in the model. The percentage of events were calculated using FREQ procedure. Data were presented as mean ± s.e.m. and percentage. *P*< 0.05 was considered significant in all analyses. All statistical analyses were conducted in SAS (SAS, Statistical Analysis System, 2012. User’s Guide, version 9.4. SAS Institute, Cary, NC.).

## Results

### Autophagy level in terms of expression of LC3 is lower in SCNT goat embryos as compared to IVF counterparts during pronucleus stage

Regarding the importance of autophagy activation during the early stages of pre-implantation development, we studied the autophagy status in terms of LC3 protein expression by immunofluorescence staining in the pronucleus stage of IVF embryos at 11, 14 and 17 hpi and SCNT embryos at 3, 6 and 9 hpaembryos (Fig 1A and 1B). Our results showed a higher number of LC3 dots,primarilyrepresenting autophagosomes, in IVF embryos at 11, 14 and 17 hpi compared to their SCNT counterparts at 3, 6 and 9 hpa (*P<*0.05, Fig 1C and 1D). importantly, there was no significant difference in the number of LC3 dots among IVF embryos at 11, 14 and 17 hpi (*P*> 0.05, Fig 1D). Incontrast, SCNT-derived exhibitedvery few LC3 dots at 3 and 6 hpa, with only a slight increase observed at 9 hpa,which was significantly lower than the number of LC3 dots in IVF embryos(Fig 1C and 1D). Theseresults demonstrate that activation of autophagy is absent during early one-cell stage (the initial hours after activation) in goat SCNT embryos.

### Rapamycin, as a mTORC1 inhibitor, elevates the autophagy level of SCNT goat embryos during the pronucleus stage

**Fig 2:**
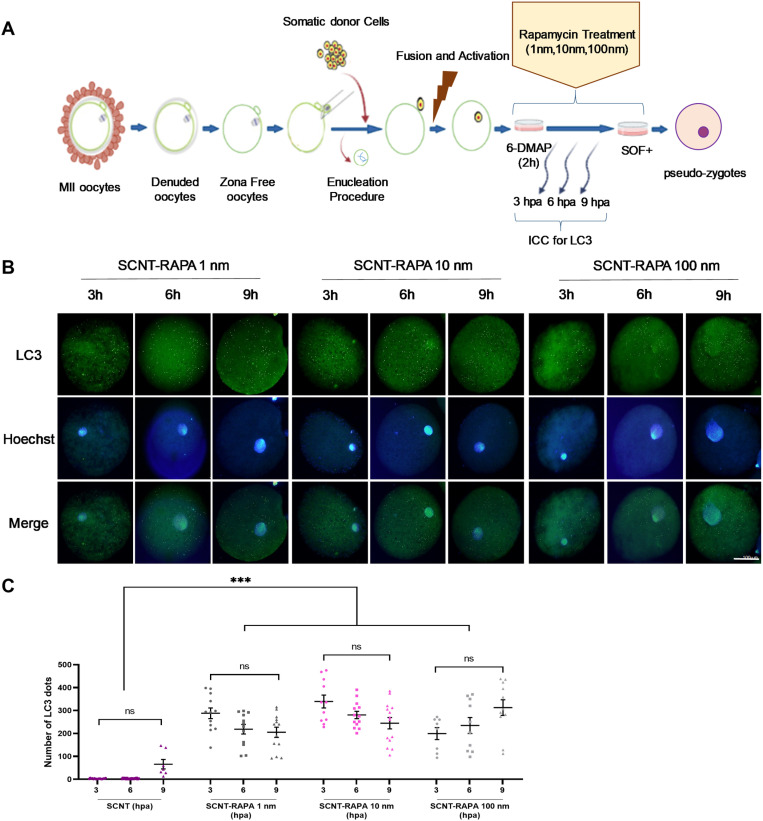
(A) A schematic review of SCNT procedures that were carried out in this study for evaluating the effect of rapamycin on expression of LC3 in pronucleus stage. (B) Expression of LC3 in the pronucleus stage of goat SCNT embryos that were treated with 1, 10 and 100 nM rapamycin after activation (3, 6 and 9 hpa). Green, LC3 labelled protein (upper row); Blue, chromatin labelled with Hoechst (middle row) and Merge, LC3 and chromatin merged (lower images). Scale bar is the same in all pictures and represents 100 μm. (C) Quantification of LC3 in SCNT goat embryos treated and non-treated with rapamycin. ***P< 0.001 and ns, non-significant.

The number of LC3 dots in SCNT embryos during the first 9 hours after activation was less than IVF embryos,indicating a lower autophagy level (Fig 1C and 1D). To further investigate this, SCNT embryos were treated with various concentrations of Rapamycin (1, 10, and 100 nM) during the first 9hours after activation (3, 6, and 9 hpa) (Fig 2A). Subsequently, the autophagy status was assessed by immunofluorescence staining of LC3 protein in the (pseudo) pronucleus stage of SCNT embryos. Interestingly, treatment of SCNT goat embryos with Rapamycin significantly increased the number of LC3 dots at 3, 6, and 9 hpa compared to non-treated counterparts (*P<*0.05, Fig 2B and 2C). The level of autophagy was similar across 3, 6, and 9 hpain the treated groups, with no significant differences between time points. Additionally, autophagy levels were slightly higher in the 10 and 100 nM Rapamycin-treated groupscompared to the1 nM treated group, but these differences were not statistically significant (*P*> 0.05, Fig 2B and 2C) (*P>*0.05, Fig 2B and 2C).

The results of this experiment demonstrate that Rapamycin can effectively stimulate autophagy in SCNT goat embryos during the early stages of development, potentially compensating for the lower basal autophagy levels observed in these embryos compared to IVF embryos.

### Autophagy induction via rapamycin treatment accelerates the developmental competence of goat SCNT embryos

**Fig 3:**
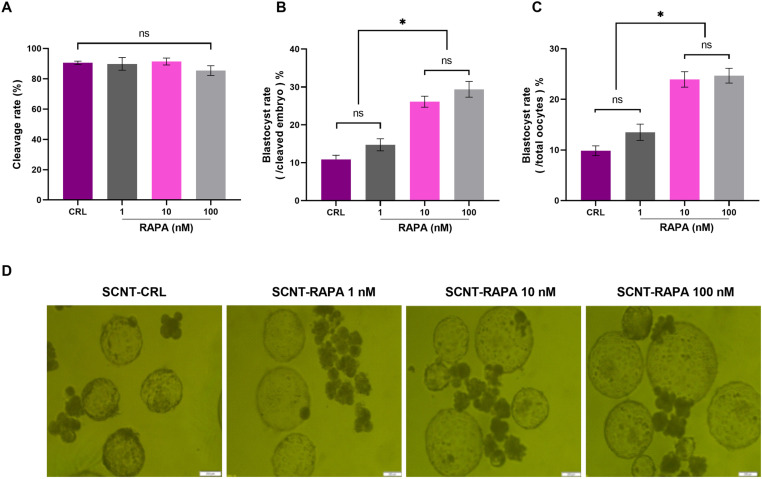
The effect 1, 10 and 100 nM of rapamycin during early hours (2 h) after activation in goat SCNT embryos on (A) cleavage rate, (B) blastocyst rate (/cleaved embryos) and (C) blastocyst rate (/total oocytes). **(D)** The representative bright-field images of blastocysts in various groups, Scale bars represent 200 μm.*P< 0.05 and ns, non-significant.

In the next step, we assessed the impact ofautophagy induction via Rapamycin treatment (1, 10 and 100 nM) on developmental competence of goat SCNT embryos,focusing oncleavage and blastocyst rates. Cleavage and blastocysts rates were assessed onday 3 and 7, respectively. As shownin Fig 3, there was no significant difference in cleavage rates among the treatment groups (*P>*0.05, Fig 3A). However,the blastocysts ratesweresignificantly higherin the 10 and 100 nM Rapamycin-treated groups compared to the control and 1 nM Rapamycin groups (*P<*0.05, Fig 3B, 3C and 3D).

These findings indicate that while rapamycin treatment did not affect cleavage rates, it significantly enhanced the developmental competence of SCNT goat embryos, as evidenced by the improved blastocyst rates in higher concentration treatments.

### Rapamycin treatment does not alter the gene expression in blastocysts

**Fig 4:**
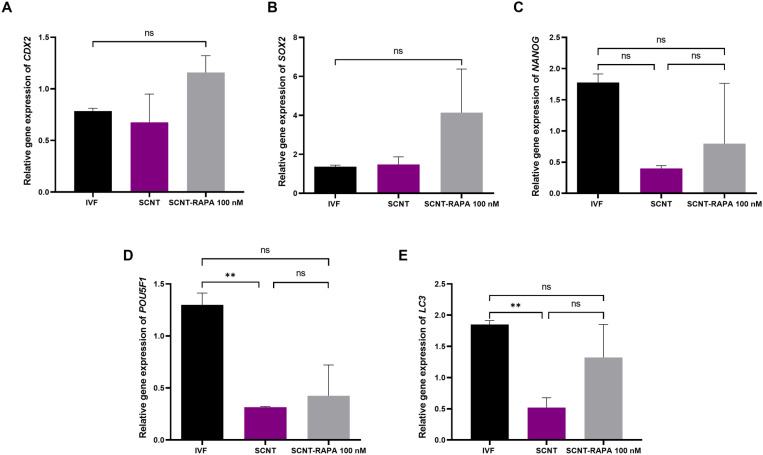
The effect 1, 10 and 100 nM of rapamycin during early hours after activation in goat SCNT embryos on the relative expression of *CDX2* (trophectodermal related genes), *LC3* (autophagy related genes), and *NANOG*, *SOX2* and *POU5F1* (pluripotency related genes). **P< 0.01 and ns, non-significant.

Results from real-time gene expression analysis are shown in Fig 4. The relative expression levels of key trophectodermal markers, such as*CDX2* (Fig 4A), as well aspluripotency-related genes including *SOX2* (Fig 4B), *NANOG* (Fig 4C) and *POU5F1* (Fig 4D), were not significantly different (*P*> 0.05) among blastocysts derived from various experimental groups. Additionally, the mRNA expression levels of *LC3* did not differ significantly among the treatment groups (*P*> 0.05, Fig 4E).

### Absence of 6-DMAP during activation of reconstructed oocytes increase the nuclear abnormality and results in the failure of blastocyst formation

**Fig 5:**
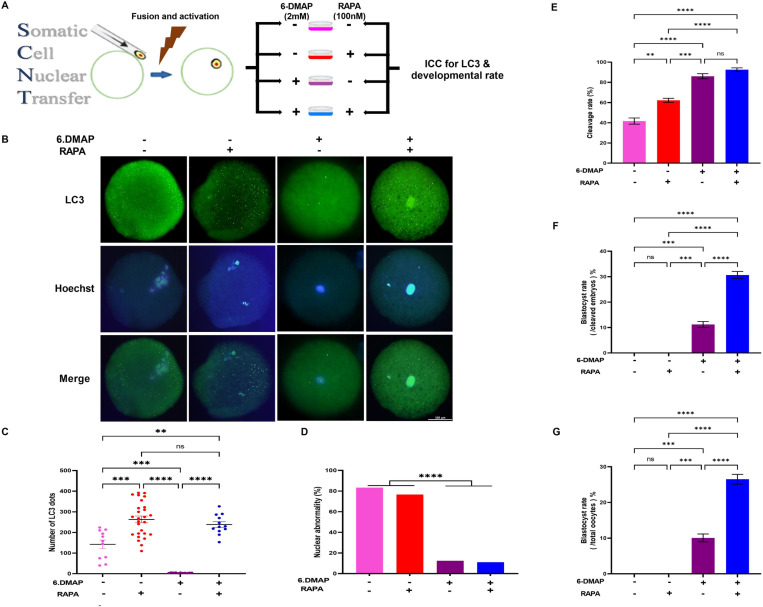
(A) A schematic review of SCNT procedures in presence or absence of 6-DMAP that were carried out in this study for evaluating the effect of 6-DMAP on expression of LC3 in pronucleus stage. (B) Expression of LC3 in the pronucleus stage of goat SCNT embryos treated and non-treated with rapamycinin presence and absence of 6-DMAP. Green, LC3 labelled protein (upper row); Blue, chromatin labelled with Hoechst (middle row) and Merge, LC3 and chromatin merged (lower images). Scale bar is the same in all pictures and represents 100 μm. (C) Quantification of LC3 in SCNT goat embryos treated and non-treated with rapamycinin presence and absence of 6-DMAP. (D) nuclear abnormality rate in SCNT goat embryos treated and non-treated with rapamycinin presence and absence of 6-DMAP. (E) cleavage rate, (F) blastocyst rate (/cleaved embryos) and (G) blastocyst rate (/total oocytes) in SCNT goat embryos treated and non-treated with rapamycinin presence and absence of 6-DMAP.*P< 0.05, **P< 0.01, ***P<0.001, ****P< 0.0001 and ns, non-significant.

6-DMAP, a MAPK inhibitor, is commonly used in SCNT techniques to maintain the diploid complement in farm animal species including bovine, sheep, and goat. To investigate whether the inhibition of MAPK activity by6-DMAP has a regulatory effect on autophagic levels during SCNT embryo activation, we designed an experiment to assessthe role of 6-DMAP on autophagy and embryo development (Fig 5A). After immunostaining forLC3, a marker of autophagosome, we observed that the number of LC3 dots was remarkably higher in SCNT embryos activated in the absence of 6-DMAP compared to those activated in its presence (*P*< 0.05, Fig 5B and 5C).However, this elevated autophagy level in the absence of 6-DMAP was accompanied by a significant increase in nuclear abnormalities (*P*< 0.05, Fig 5D). Thesefindings align with previous reports that identify 6-DMAP as a crucial component in SCNT techniques, necessary for maintaining normal diploidy in embryos of farm animal species. Consistent with the increased nuclear abnormalities observed in the absence of 6-DMAP, our data revealed a significantly lower cleavage rate, both in the absence and presence of rapamycin (*P*< 0.05, Fig 5E). Furthermore, embryos with lower cleavage rates in the absence of 6-DMAP did not progress to the blastocyst stage (*P*< 0.05, Fig 5F and 5G).

These results highlight the importance of 6-DMAP in regulating autophagy levels and maintaining developmental competence in SCNT embryos. By inhibiting MAPK activity, 6-DMAP helps preserve normal diploidy, which is essential for successful embryonic development.

### Treatment of one-cell stage SCNT goat embryos with 6-DMAP reduces the autophagy level by lower ratio of p44/42 MAPK/βACTIN

**Fig 6:**
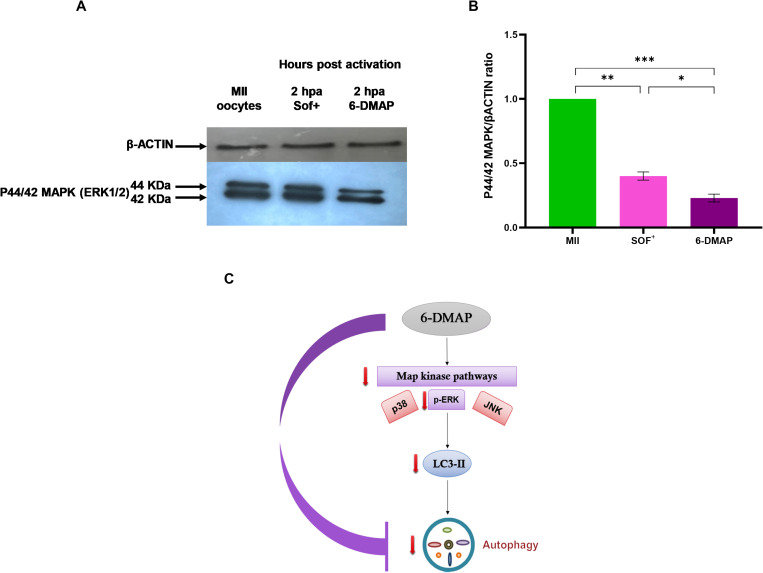
(A) Western blotting assay for detection of the expression level of p-ERK1/2 in MII oocytes, and SCNT goat embryos in absence and presence of 6-DMAP. (B) Quantification of Western blot bands at 42 and 44 kDa for pERK1 and pERK2, respectively, on the blots that are shown in A part. (C) A schematic diagram that shows how 6-DMAP can inhibit autophagy through inhibition of phosphorylation of ERK1/2. *P<0.05, **P<0.01 and ***P< 0.001.

To further investigate the mechanism by which 6-DMAP decreases autophagy levels, we assessed the level of phosphorylated ERK1/2 (phosphor-ERK1/2) protein usingwestern blot analysis. It has been shown that inhibition of ERK1/2 phosphorylation preventsthe conversion of LC3-I to LC3-II, thereby decreasingautophagy status (Fig 6C). Interestingly, we observed that the expression of phospho-ERK1/2 in absence of 6-DMAP was significantly higher than in its presence (*P*< 0.05, Fig 6A and 6B, S1 and S2 Fig). Our results indicate that the decrease in autophagy levels during the one-cell stage in goat SCNT embryos is caused by 6-DMAP through the inhibition of ERK1/2 phosphorylation (Fig 6).

This finding underscores the role of ERK1/2 signaling in regulating autophagy and suggests that the modulation of this pathway may be crucial for optimizing developmental outcomes in SCNT embryos. Understanding this mechanism may provide insights into improving embryo viability in reproductive technologies.

## Discussion

During early embryo development, the degradation of maternally-inherited mRNA and proteins is essential for successful MET and EGA in both mammalian and also non-mammalian species. The primary mechanisms contributingtothe degradation of mRNA and proteins are the ubiquitin-proteasome system (UPS) and autophagy. Severalstudies have demonstratedthat autophagy is extensively activated during the process of MET [49, 50]. Additionally, Song et al. indicated that the autophagy pathway isconstitutively activated and playsa fundamental role in early human embryo development [39]. In this context, ongoing researches are investigating the essential roles of autophagy during MET and EGA events, as well as its regulating functions during early embryogenesis.Understanding these processes is crucial for elucidating the mechanisms that govern embryonic development and may have implications for improving reproductive technologies and developmental outcomes in assisted reproductive techniques.

In this study, we first identified that autophagy activation, indicatedby immunofluorescent staining of LC3, is absent in the pseudo-zygote stage of SCNT embryos at 3 and 6 hpa compared to their IVF counterparts at 11 and 14 hpi, respectively. Notably, the autophagy protein,LC3, was firstobserved at 9 hpa in a significantly lower level compared to theIVF counterparts at 17 hpi. Our results are consistent with a previous study in which it was demonstrated that no autophagosome formation (defined by co-immunostaining of LC3 and p62)was detected during the first 6 h of activation in mouse SCNT embryos [51]. We should mention that while co-immunostaining with p62 is indeed a valuable approach to further validate autophagy measurements, we were unable to perform this due to resource limitations in our laboratory. Understanding the dynamics of LC3 and p62 is crucial, as LC3 is a marker of autophagosomes, while p62 is involved in the degradation of ubiquitinated proteins, both of which are essential for assessing autophagy levels accurately.

To compensate for the lowautophagy levels in goat SCNT embryos, the reconstructed oocytes weretreated with various concentrations of rapamycinimmediately after chemical activation with ionomycin. Rapamycin, an mTORC1 inhibitor, is well known for its ability to increase autophagy. Remarkably, we observed that the number of LC3 dots, which serve as markers ofautophagosomes, increased during the early hours after activation (3, 6, and 9 hpa) to a level similar to that of IVF-derived zygotes. These results areconsistent with the findings of Shen et al., who demonstrated the restoration of autophagy levelsusingtwo chemical inhibitors of mTORC1,Rapamycin and PP242, in mouse pseudo-pronucleus stage SCNT embryos.Additionally, Song et al. showed that LC3-II levels and dot-type LC3 immunoreactivity were greatly increased by the treatment of 2-cell bovine embryos (IVF-derived) with rapamycin, indicating the successful activation of autophagy by this compound [52]. The ability of rapamycin to induce autophagy in SCNT embryos is crucial, as it may help compensate for the inherently low autophagy levels observed in these embryos compared to their IVF counterparts. By stimulating the formation of autophagosomes, rapamycin can potentially enhance the degradation of maternally-inherited mRNA and proteins, which is essential for successful maternal-to-embryonic transition and embryonic genome activation.

In addition to the commonly used chemical inhibitors of mTORC1, such asrapamycin and PP242, there are other compounds including chaetocin, IL1-β, melatonin, trehalose, and vitamin C, that are not primarily recognized for their autophagy-inducing properties. However,severalstudies have demonstrated their potentialto induce autophagy [53–56]. Based on these findings, additional research has explored the effects of chaetocin, IL1-β, melatonin, trehalose and vitamin C autophagic activity in preimplantation embryos across various species, including bovine, ovine, porcine, and mouse [57–61]. These studies have observed beneficial effects following autophagy induction on preimplantation embryo development.

In contrast to our and also previous results (Shen et al, Song BS, et al., Chi et al) [51,52,62], previously it was reported that fertilization-induced activation of autophagy is not dependent on mTORC1 [50]. In a previous study,it was shown that Torin1 andPP242a, specific mTORC1 inhibitors, decreased mTORC1 activity but did not increase autophagy level. In the current study, we observed the induction of autophagy in one-cell stage SCNT embryos following treatment with Rapamycin, an inhibitor of mTORC1. Additionally, goat SCNT embryos exhibited improved blastocyst rates due to autophagy compensation resulting from Rapamycin treatment. Our findings suggest that different mechanisms may regulate autophagy in SCNT- and IVF-derived embryos. Furthermore, these differences could be related to species-specific factors, particularly when comparing mouse and goat embryos.In this study, we evaluated whether enhancing autophagy levelsduring the early one-cell stagecould improve the developmental competence of goat SCNT embryos. Our results indicate that treatment with 100 nM rapamycin significantly increased the number of LC3 dots, a marker of autophagy, compared to the control group. This finding is consistent with the established role of rapamycin as an mTORC1 inhibitor, which activates autophagy [63]. Both the 10 nM and 100 nM concentrations of rapamycin wereeffective in elevating autophagy levels in SCNT embryos during the pronucleus stage. Notably, both the 10 nM and 100 nM Rapamycin treatment groups demonstrated a significant improvement in the blastocyst rate compared to the control group. These results underscore the potential benefits of autophagy induction via rapamycin treatment in enhancing the developmental competence of SCNT embryos. The improved blastocyst rate observed with both 10 nM and 100 nM Rapamycin suggests that these concentrations are effective for promoting embryo development without adversely affecting gene expression.However, it is important to note that gene expression analysis was conducted at the blastocyst stage, and we did notassess the effects of 10 nM and 100 nM Rapamycin on earlier developmental stages or other quality metrics of the embryos. Future studies should explore these aspects to provide a more comprehensive understanding of how rapamycin treatment influences SCNT embryo quality throughout development.

The role of autophagy during fertilization and early embryonic development is under intense investigation. While a series of studies investigated the role of autophagy in fertilized embryos, very fewstudies areavailable onSCNT embryos. Tsukamoto and colleagues reported that the level of autophagy is low in unfertilized oocytes; however, autophagy is activated immediately after fertilization [13]. This activation of autophagy after fertilization appears to play a critical role during early embryonic development in mouse species. Severalstudies have shown that autophagy becomes active immediately after fertilization and remains active until the late zygote stage in mouse species. For a brieftransition period, autophagy becomes temporarily inactive until the middle of 2-cell stage, after which it is reactivated until the 8-cell stage. Followingthis stage,autophagy gradually decreases until it reaches basal levels during the implantation period [33,37,38].

It has been shown that repression of autophagy using 3-MA (an autophagy inhibitor) decreased the blastocyst yield in mouse and bovine species [49]. Notably, these studies have shown that synthesis of proteinis limited in the autophagy-deficient embryos which indicate that degradation of maternally inherited proteins through autophagy is a critical event during preimplantation development [13].

As we mentioned earlier, Shen et al. showed that rapamycin restored reprogramming efficiency and embryonic development in mouse SCNT embryos. In addition, Pan et al. demonstrates that exogenous IL-1β can effectively stimulate autophagy in mouse embryos at the 2-cell, 4-cell, 8-cell, and blastocyst stages, andadditionally, helps to improve the quality of blastocysts.

In addition to the role of autophagy in the embryonic development of mouse species, a series of studies have investigated its significancein mammalian farm animals.With regard to this, it has been shown that the transient activation of autophagy pathway in preimplantation bovine embryos increases blastocyst yield. Furthermore, these authors demonstrated that elevatedautophagy levels can reduce endoplasmic reticulum stress in preimplantation embryos [52,57,64]. Balboula and colleagues reportedhigher autophagic activity in good-quality IVF embryos compared to poor-quality IVF embryos in bovine species [64]. Additionally, in another study, Li et al. demonstrated the rescue effect of autophagy induction onthe developmental competence of poor-quality COCs [65]. Furthermore, Sugiyama et al. have shown that the quality of oocytes from early antral follicles of aged cows improved after supplementation of the culture medium with resveratrol,whichinducedautophagy and mitochondrial biogenesis [66].

Several studies investigated the role of autophagy and its induction on preimplantation development in porcine species. It has been shown that Rapamycin treatment (1, 10, and 100 nM) positively influences the preimplantation development of porcine embryos, including both parthenogenetic and IVF embryos, likely by modulating the cellular redox state and promoting autophagy [67]. Additionally, bothhigh and low concentrations of oxygen (21% and 1%, respectively) inhibitautophagy in embryos and reducethe developmental competence of parthenogenetic porcine embryos [68, 69]. These findings underscorethe importance of autophagy activation for proper embryonic development in porcine species.Notably, these studies demonstrated that the induction of autophagy through rapamycin treatmentrestored the developmental parameters of parthenogenetic embryos cultured under 20% and 1% oxygen tension, aligning them with the levels observed in standard culture conditions. Thisindicates that the early development of porcine embryos is influenced by the interplay between oxygen tension and autophagy [68, 69]. In another study, Cai et al. suggested that treatment with trehalose during IVM enhances the developmental potential of porcine embryos by downregulating pro-apoptotic genes and upregulating autophagy-related genes and markers [58]. Furthermore, in one of our recent studies, we demonstrated that melatonin promotes autophagy in ovine SCNT embryos, as indicated by a higher number of LC3B dots, and improvesthe preimplantation development of these embryos [59].

Finally, in addition to these studies on farm animal species, Adel et al., highlighted autophagy as a tightly regulated process that is essential for maintaining cell allocation and differentiation during the later stages of human preimplantation embryo development [70]. Collectively, these studiesdemonstrate a strong association between autophagy and the developmental characteristics of growing embryos, presenting new approaches for the production of high-quality blastocysts, particularly *in vitro*-derived ones.

The developmental defects of SCNT embryos primarily occurduring the EGA phase, which happensat the 2-cell stage in mice, at the 4- to 8-cell stage in goats, pigs and humans, and at the 8- to 16-cell stage in sheep and cattle [3–8]. In this study, we demonstrated that goat SCNT embryos exhibit abnormal levels ofautophagy compared to IVF embryos. Furthermore, it has been shown that the EGA phase can be significantly influenced by autophagy levels in porcine SCNT embryos [62]. Chi et al. demonstrated that the type of embryo (IVF vs. SCNT vs. PA) affects autophagy status. In this regard,the highest autophagy levelswereobserved in the zygote stage of IVF-derived embryos, while similar levels were noted at the 2-cell stage in SCNT and at the 4-cell stage in PA embryos [35,36,52,62,71]. These results indicate a delay in autophagy activation, which may postpone EGA and subsequently affect early embryo development. Interestingly, Masala and colleagues [72] showed a consistent delay in maternal transcript degradation in embryos derived from prepubertal oocytescompared to those from adult oocytes. This observationsuggests thatthe delay in EGA inembryos derived from prepubertal donors may lead to altereddevelopmental kinetics.

To investigate the mechanism dysregulatingautophagy in SCNT-derived embryos, we cultured the reconstructed oocytes immediately after activation in the presence or absence of 6-DMAP. Interestingly, we observed that autophagy in 6-DMAP-treated embryos waslower than in non-treated embryos. We hypothesized that 6-DMAP,a kinase inhibitor [73, 74], may inhibit the phosphorylation of ERK1/2, leading to decreasedautophagy in 6-DMAP-treated embryos. Western blot analysis revealed that the intensity of phospho-ERK1/2 waslower in 6-DMAP-treated embryos compared to non-treated ones. Remarkably, in a previous study,lysates of MII-arrested oocytes, or oocytes incubated for 1.5 or 2.5 h after activation in control medium or in 6-DMAP were analyzed by immunoblotting using an anti-ERK antibody [75]. Their results showed that phospho-ERK1/2 levels were reduced by 30% and 45% after 1.5 and 2.5h treatments with 6-DMAP, respectively. They concluded that 6-DMAP was able to moderately accelerate MAP kinase inactivation, likelyby inhibiting upstream kinases, which is consistent with our results [75].

Previous studies have shown that ERK1/2, a downstream kinase of the MAPK pathway, regulates the expression and activity of apoptosis and autophagy [76–78]. It is wellknown that inhibition of ERK1/2 phosphorylation leads to the cleavage of caspase-3 and a reduction in the expression of Bcl-2, ananti-apoptotic gene, which ultimately induces DNA fragmentation and apoptosis. Additionally, inhibition of ERK1/2 phosphorylation decreases the expression of *Beclin-1*, whichimpedes the conversion of LC3B-I to LC3B-II and subsequently affects autophagosome formation [76–78]. Interestingly, in this study, we observed that 6-DMAP decreased the LC3 dots, as detected by immunostaining in pseudo-zygote embryos, confirming the findings of previous studies.Additionally, using a control artificial activation consisting of ionomycin followed by incubation in the presents of cycloheximide instead of 6-DMAP would beinteresting and have potential value in further elucidating the mechanisms of autophagy following 6-DMAP treatment. Unfortunately, our laboratory is currently set up to use 6-DMAP, which has consistently yielded better outcomes in SCNT. Therefore, we did not include cycloheximide in our experiments and it is a limitation of our study.

Finally, we assessed the impact of autophagy activation in SCNT embryos on the quality of derived blastocysts. Results from real-time gene expression analysis did not show any significant difference inthe expression of analyzed genes (*CDX2*, *NANOG*, *POU5F1*, *SOX2, and LC3)* betweenblastocysts derived fromrapamycin-treated and non-treated SCNT embryos. In contrast to our results, previous studies have revealed that the triad of OCT4, NANOG and SOX2 can be regulated by autophagy levels [79], highlighting the importance of autophagy in regulating early embryogenesis. However, further assessments need to be carried out toevaluate the quality of the resultant embryos, including their post-implantation development.

## Conclusion

In conclusion, we demonstrated that the inhibition of phosphorylation of ERK1/1 by 6-DMAP treatment during the activation period (early hours of one-cell stage) suppressed the expression of the autophagy-related protein, LC3 in goat SCNT embryos. This reduction in autophagosome formation may account for the lower developmental competence of SCNT embryos compared toIVF embryos. In addition, our results revealed that increasing the autophagy levelthroughRapamycin treatment could improve SCNT efficiencyby approximately 2-fold in terms of blastocyst rate, with statistical significance indicating the robustness of this finding. Our findings suggest that the induction of autophagy during the early hours of the one-cell stage plays an important role in reprogramming and may be crucialfor the post-implantation development of goat SCNT embryos.Finally, while we propose that exposure to 6-DMAP contributes to reduced autophagy levels in one-cell stage SCNT embryos, we believe that a more intricate interplay of factors and mechanism influencing autophagy in these embryos may exist. Understanding these mechanisms is vital for improving the efficiency of SCNT and enhancing the viability of embryos in reproductive technologies. Further research is needed to elucidate the specific pathways involved in this process. intricate

## Supporting information

S1 Table List of primers used in this study for real time PCR. (DOCX)

S1 Fig Raw images of western blot for P44/42 MAPK (ERK1/2)used inFig6A(bottom panel) before cropping. (TIF)

S2 Fig Raw images of western blot for β-ACTIN used inFig6A (top panel) before cropping. (TIF)
