# Supplementary material for: Induction of autophagy in one-cell stage somatic cell nuclear transfer embryos improves preimplantation embryonic development in goat species
Source: PLoS One. 2025 Apr 28;20(4):e0314176. doi: 10.1371/journal.pone.0314176 (PMC12036934; doi:10.1371/journal.pone.0314176)
Supplement: S1 Table — (DOCX) [file pone.0314176.s001.docx]

**S1 Table.** List of primers used in this study for real time PCR

| **Symbol Gene** | **Forward Primer** | **Reverse Primer** | **Annealing Temp. (°C)** | **Length** |
| --- | --- | --- | --- | --- |
| *β-ACTIN* | CCATCGGCAATGAGCGGT | CGTGTTGGCGTAGAGGTC | 57 | 146 |
| *CDX2* | CCCCAAGTGAAAACCAG | TGAGAGCCCCAGTGTG | 56 | 144 |
| *LC3B* | GCCTTCTTCCTGTTAGTG | AATCCATCTTCATCCTTCTC | 54 | 144 |
| *NANOG* | GATTCTTCCACAAGCCCT | TCATTGAGCACACACAGC | 53 | 137 |
| *OCT4* | GGAAAGGTGTTCAGCCA | ATTCTCGTTGTTGTCAGC | 57 | 123 |
| *SOX2* | ATGGGCTCGGTGGTGA | CTCTGGTAGTGCTGGGA | 55 | 182 |
