# Supplementary figures and images for: Induction of autophagy in one-cell stage somatic cell nuclear transfer embryos improves preimplantation embryonic development in goat species
Source: PLoS One. 2025 Apr 28;20(4):e0314176. doi: 10.1371/journal.pone.0314176 (PMC12036934; doi:10.1371/journal.pone.0314176)

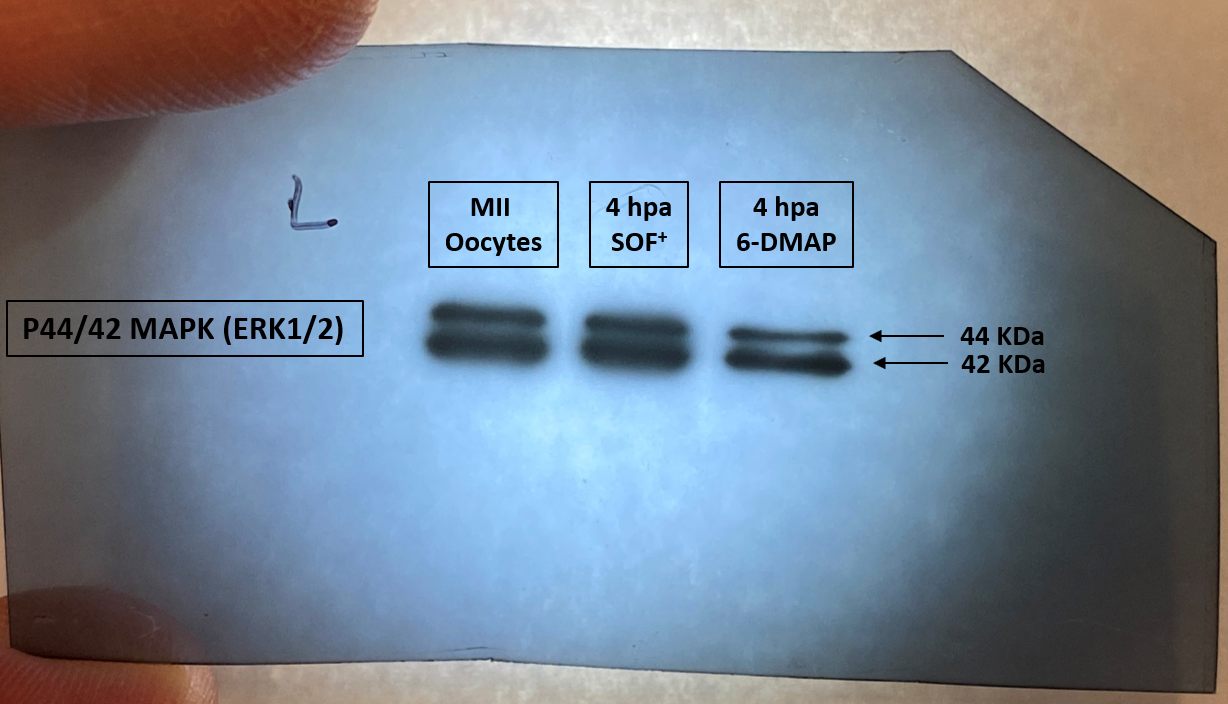

Supplement: S1 Fig — (TIF) [file pone.0314176.s002.tif]

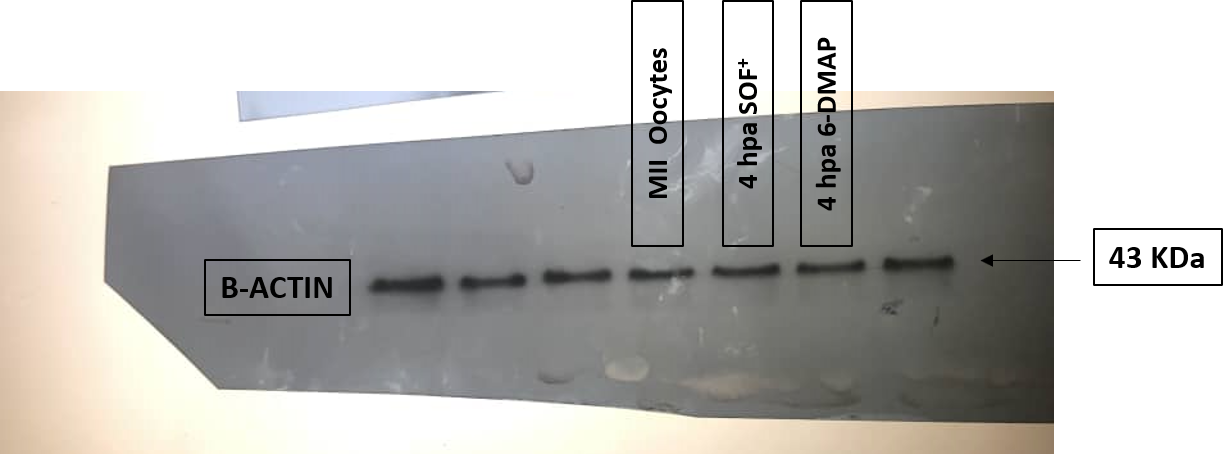

Supplement: S2 Fig — (TIF) [file pone.0314176.s003.tif]
